# Exploring the problems and coping strategies of pharmacy internship in large general hospitals in China: from the perspective of preceptors

**DOI:** 10.1186/s12909-024-05032-x

**Published:** 2024-01-17

**Authors:** Xiaojing Lu, Wan Zhang, Xuedong Jia, Xiaoyue Bao, Xiaojian Zhang, Jian Kang, Shuzhang Du, Zhao Yin

**Affiliations:** 1 Department of Pharmacy, The First Affiliated Hospital of Zhengzhou University, Zhengzhou, 450052 China; 2 Henan Drug Clinical Comprehensive Evaluation Center, Zhengzhou, 450052 China; 3 Institute for Hospital Management of Henan Province, Zhengzhou, 450052 China

**Keywords:** Hospital pharmacy preceptor (HPP), Experience, Challenges, Strategies, Qualitative study

## Abstract

**Objective:**

The role of the Hospital Pharmacy Preceptor (HPP) is pivotal in upholding the excellence of experiential training and fostering the professional growth of pharmacy interns. However, there is a lack of studies that provide an overview of pharmacy internships from the perspective of HPP. This study explores the experience and expectations of HPPs regarding existing problems and possible coping strategies in intern teaching.

**Methods:**

This is a qualitative study that was conducted through individual interviews and focus group discussions. HPPs were invited as participants from large-scale tertiary hospitals in representative provinces of mainland China. Interview and focus group discussion data were analyzed using thematic analysis to see emerging themes from the data. Nvivo 12 was utilized for data management and processing.

**Results:**

Eight individual interviews and two focus group discussions were conducted, involving 14 HPPs as participants. Upon the examination of the interviews and focus group data, four themes were summarized regarding HPPs’ perceptions: 1) current presenting problems; 2) possible coping strategies; 3) something HPPs should do; 4) something interns should do.

**Conclusion:**

This study found that from the HPPs’ perspective, the hospital-based pharmacy internship still has some problems from policy to practice, which need to be addressed by the joint efforts of the state, schools, internship bases, pharmacy preceptors, and students.

**Supplementary Information:**

The online version contains supplementary material available at 10.1186/s12909-024-05032-x.

## Introduction

With an increasing emphasis on comprehensive pharmaceutical care [1, 2], the roles of pharmacists have undergone significant evolution and expansion within the current framework of patient-centered care. As the role of pharmacists changes, the requirement for high-quality practice-ready pharmacy graduates increases and as a significant part of undergraduate pharmacy education, pharmacy internship becomes increasingly important [3, 4]. By completing a certain period of pharmacy internships in a pharmacy department at a qualified teaching hospital, pharmacy students can gain a practical understanding of drug dispensing, drug-management concepts, drug consultation, clinical pharmacy services, etc., to get a broader view of the pharmacy profession [5]. For example, in the European Union (EU), a pharmacist is a person who has completed a curriculum in university-level pharmaceutical education, including 6-month pharmacy internship, which provides adequate preparation for the unsupervised, full-responsibility provision of pharmacy services in a pharmacy setting [6]; In the USA, the Accreditation Council for Pharmacy Education requires pharmacy students to complete 300 introductory pharmacy practice experience hours before they start their advanced pharmacy practice experience [7]; In China, for most undergraduate pharmacy programs, each college has different curriculum setting, but generally include general education courses, foundation specialty courses, professional and service courses, research and individual development courses, and some element of practical experience, such as pharmacy medical practice. Among them, pharmacy students are required to attend the pharmacy practice during the final year of their degree, and the time ranges from 6 months to 1 year [1].

Compared to other countries, hospital-based pharmacy internships in China started late, with a history of only 30 years [8]. Yi et al. explored the differences between U.S. and Chinese pharmacy education, the study noted that, in clinical practice, U.S. pharmacy students show proficiency in drug dosages and interactions when reviewing drug orders, can propose medication recommendations for patients according to their symptoms, can add their ideas to physicians’ treatment plan and can skillfully search the relevant databases when they encounter problems in their internship. While most Chinese pharmacy students are not as proficient in these areas [9]. To reduce this disparity, hospital pharmacy practice in China continued to learn from developed countries, in some teaching hospitals gradually formed a relatively mature project [8, 10]. As reported, Peking University Third Hospital started United States-China international pharmacy education programs(IPEP), a type of cultural exchange curriculum, in China in 2008 [8]. A follow-up study confirmed that the scores of overall IPEP evaluation were high, suggesting high satisfaction with the international programs [10]. However, international cooperation is only one approach and is not easily replicated. We still need to explore hospital pharmacy internships that are appropriate for our country. Many studies have confirmed that interns’ perceptions and opinions are important references for the arrangement of educational programs [11, 12], and collaboration between interns and hospital pharmacy preceptors (HPP) at internship hospitals can create effective educational programs [13].

Our previous qualitative study explored the experiences and expectations of pharmacy interns from their perspective and identified problems with current pharmacy internships in mainland China, such as a lack of nation-level operational guidelines; no bidirectional evaluation tool for evaluating the effectiveness of internships; and the training content of the hospital-based pharmacy internship is not well integrated with the existing education activities, etc. [10]. These problems tend to affect the experiences and gains of pharmacist interns. It is vital to identify the problems for the subsequent proposal of targeted solutions. However, the internship is a process in which students and teachers cooperate, and both protagonists are crucial [14]. Therefore, comprehensive information may not be obtained only from the perspective of interns, and we also need to discuss it from the perspective of teachers. However, to the best of our knowledge, there have not been any studies that provide an overview of pharmacy internships from the perspective of HPP. It is essential to gain insights into the sentiments of HPPs based on their own experiential teaching experiences and interactions with pharmacy interns.

Based on a comprehensive analysis of the relevant literature, we conducted the current qualitative study based on the previous research of our group. HPPs from large-scale tertiary hospitals in representative provinces of mainland China were invited as participants. By exploring the existing problems in interns teaching and possible coping strategies, and drawing out what specifically the HPPs and students should do from HPPs’ perspective to provide references for future hospital pharmacy education reform in China or other countries.

## Methods

### Design

This is a qualitative study that was conducted through individual interviews and focus group discussions with HPPs in China. Individual interview is the most common format of data collection in qualitative research [15]. It is useful when a researcher works with a complex issue because he can use spontaneous questions to explore, deepen understanding, and clarify answers to questions [16]. But it also has drawbacks, such as the hierarchical position and the power of the interviewer over the participants and the lack of group dynamics [17]. The focus group discussion relies on the dynamic interaction between participants to generate data that would be impossible to collect via other methods, such as individual interviewing [18]. Since, for some participants, it could be easier to disclose personal sensitive information through individual interviews, but for others, the focus group discussion could be more appropriate [19]. Therefore, we conducted both individual interview and focus group discussion. The study is reported following the Consolidated Criteria for Reporting Qualitative Studies (COREQ) checklist [20] (Table S1).

### Ethical consideration

Ethical approval for the study was obtained from the First Affiliated Hospital of Zhengzhou University Institutional Review Board (No. 2022-KY-0736). Written informed consent was obtained from all the study participants.

### Sampling and recruitment

Purposeful sampling was used to obtain participants that represented the spectrum of respondent characteristics. To meet inclusion criteria, individuals had to be hospital pharmacy preceptors who participated in the work of interns teaching and were willing to share their views and work experiences. Three large teaching hospitals in representative provinces of mainland China were selected. The sample hospitals were the First Affiliated Hospital of Zhengzhou University (approximately 8500 beds and 400 pharmacists), Sichuan Provincial People's Hospital (approximately 4300 beds and 280 pharmacists), and the First Affiliated Hospital of Zhongshan University (approximately 3500 beds and 158 pharmacists). These hospitals are traditional teaching hospitals for medical students including pharmacy students. The hardware conditions available in these hospitals for students include pharmacy (outpatient pharmacy, inpatient pharmacy, and emergency pharmacy), pharmacy intravenous admixture services centers, applied and translational centers of precision clinical pharmacy, and clinical pharmacology centers, providing pharmacy interns with diverse internship options.

### Data collection

Due to the restrictions of the COVID-19 pandemic, interviews with HPPs from Sichuan Provincial People's Hospital and the First Affiliated Hospital of Zhongshan University were conducted in the form of focus group discussions via video conference, each group had three HPPs. Interviews with HPPs from the First Affiliated Hospital of Zhengzhou University were conducted through face-to-face individual interviews. Before the formal interviews, demographic data were collected using the online platform Questionnaire Star (Changsha Ranxing Information Technology Co., Ltd), a widely used free online questionnaire platform in China, then, the researchers communicated with HPPs about the purpose of the study, the interview format, and the time for interviews, emphasizing that the participant could choose to discontinue the interview at any time. All interviews took place from December 2021 to June 2022 conducted by members of the research team consisting of clinical pharmacists and pharmacy directors with qualitative research experience. With participants’ consent, all interviews were digitally recorded and transcribed. The same interview outline, used for the focus group and one-on-one interviews, designed by ZY and WZ concretely included the following items: 1)What do you think are the characteristics of the best-performing interns? 2)Do you have any impressions of the poorly performing interns you have supervised? What are their characteristics? 3)What do you think are the biggest problems with the current pharmacy internship? 4)What would you suggest to improve the current practice? Based on the completion of two focus groups discussions, data saturation was reached after completing an individual interview with 8 HPPs, which was defined as “no new themes or codes emerging from interviews”. All interviews were conducted in mandarin, the interview outline and the selected citations were translated into English when writing the manuscript.

### Data analysis

**Fig. 1:**
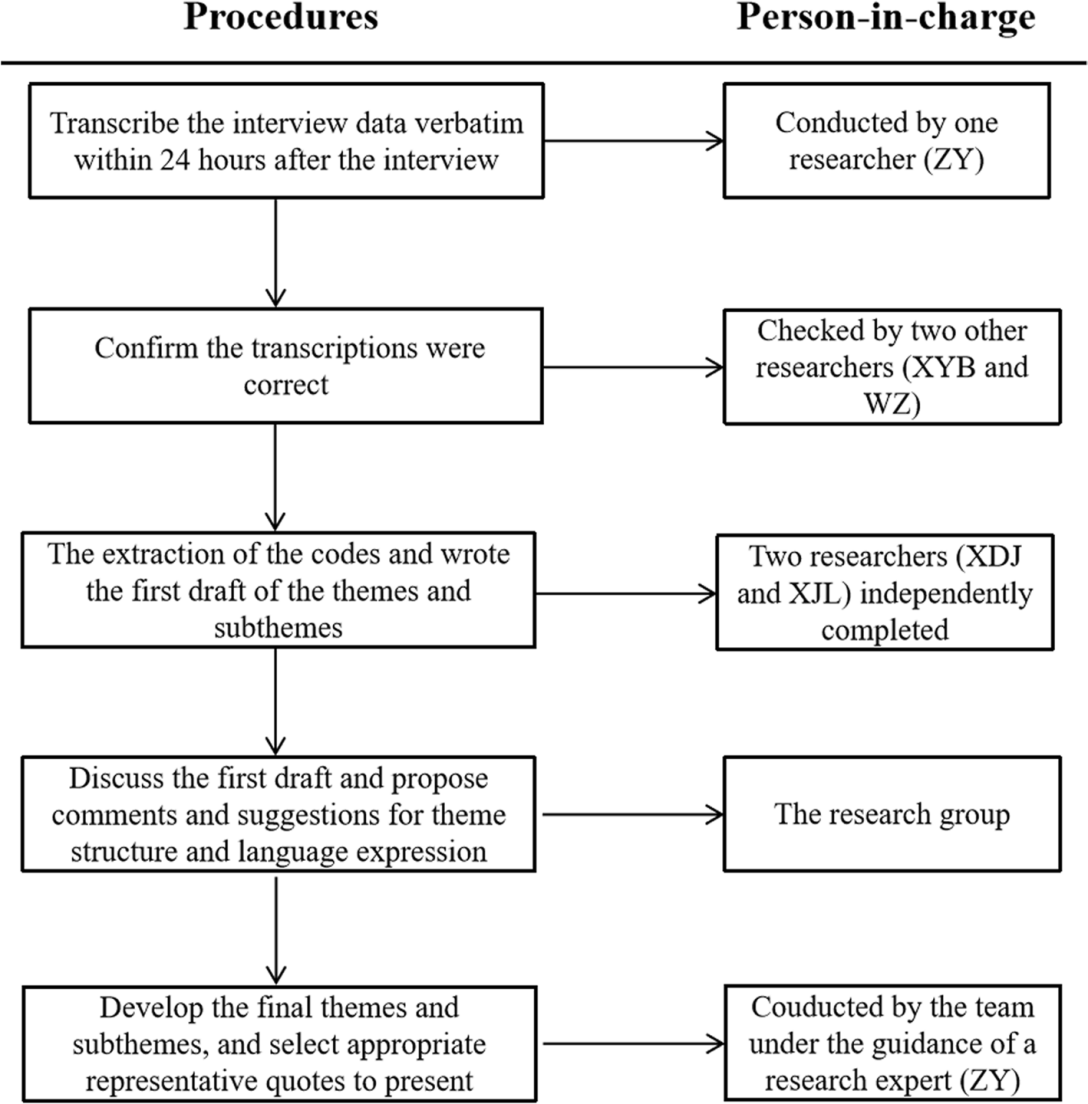
Flow chart for thematic analysis

Interview data were analyzed using a thematic analysis approach, which is a method for identifying, analyzing, and reporting patterns within data that is widely used in qualitative research [8, 21]. The steps of the thematic analysis in this study and the corresponding person-in-charge are shown in Fig. 1. In the process, NVivo12 software was used to manage and analyze the data.

### Trustworthiness

We took measures to ensure the trustworthiness of this study as described in our previous study [8, 22].

## Results

### Demographic characteristics and teaching experience of HPPs

A total of 14 HPPs were included in this study, their demographic characteristics and teaching experience are shown in Table 1. Most of the participants (*n* = 9) were female, the mean age was 36.9 years old (SD = 7.0), and the average time working as a hospital pharmacist was 12.2 years (SD = 8.5). All had a bachelor’s degree or higher, as well as an intermediate professional post or higher. 92.9% of participants expressed they are familiar with the pharmacy internship. The average number of interns they have supervised is 133.1, the frequency of discussing issues with interns is 3.1 times per week, and the frequency of summarizing the internship with interns is 3 times per week.

**Table 1:** Demographic characteristics and teaching experience of HPPs involved in the qualitative studies (*n* = 14)

Demographic characteristics	Value
**Gender**	
Male	5
Female	9
**Age, years**	
Mean ± SD	36.9 ± 7.0
**Years working as a hospital pharmacist**	
Mean ± SD	12.2 ± 8.5
**Hospital grade**	
Grade IIIA^a^	14
**Educational level**	
Bachelor	5
Master	7
Doctorate	2
**Professional title**	
Intermediate professional post	10
Associate senior professional post	4
**Familiarity with the internship**	
Very familiar	6
Familiar	7
General familiar	1
**The number of interns supervised**	
Mean ± SD	133.1 ± 248.4
**Frequency of discussing issues with interns per week**	
Mean ± SD	3.1 ± 1.8
**Frequency of summarized the internship with interns per week**	
Mean ± SD	1.4 ± 0.5

^a^The highest level of hospital in China

### Qualitative results

In our study, we combined individual interviews and focus group discussions to overcome each method’s weaknesses and limitations. Some researchers have argued that multiple-method research has some challenges, one is how to address the findings of two methods if they are not complementary but conflicting. Fortunately, data gathering through these two methods is complementary in our study.

**Fig. 2:**
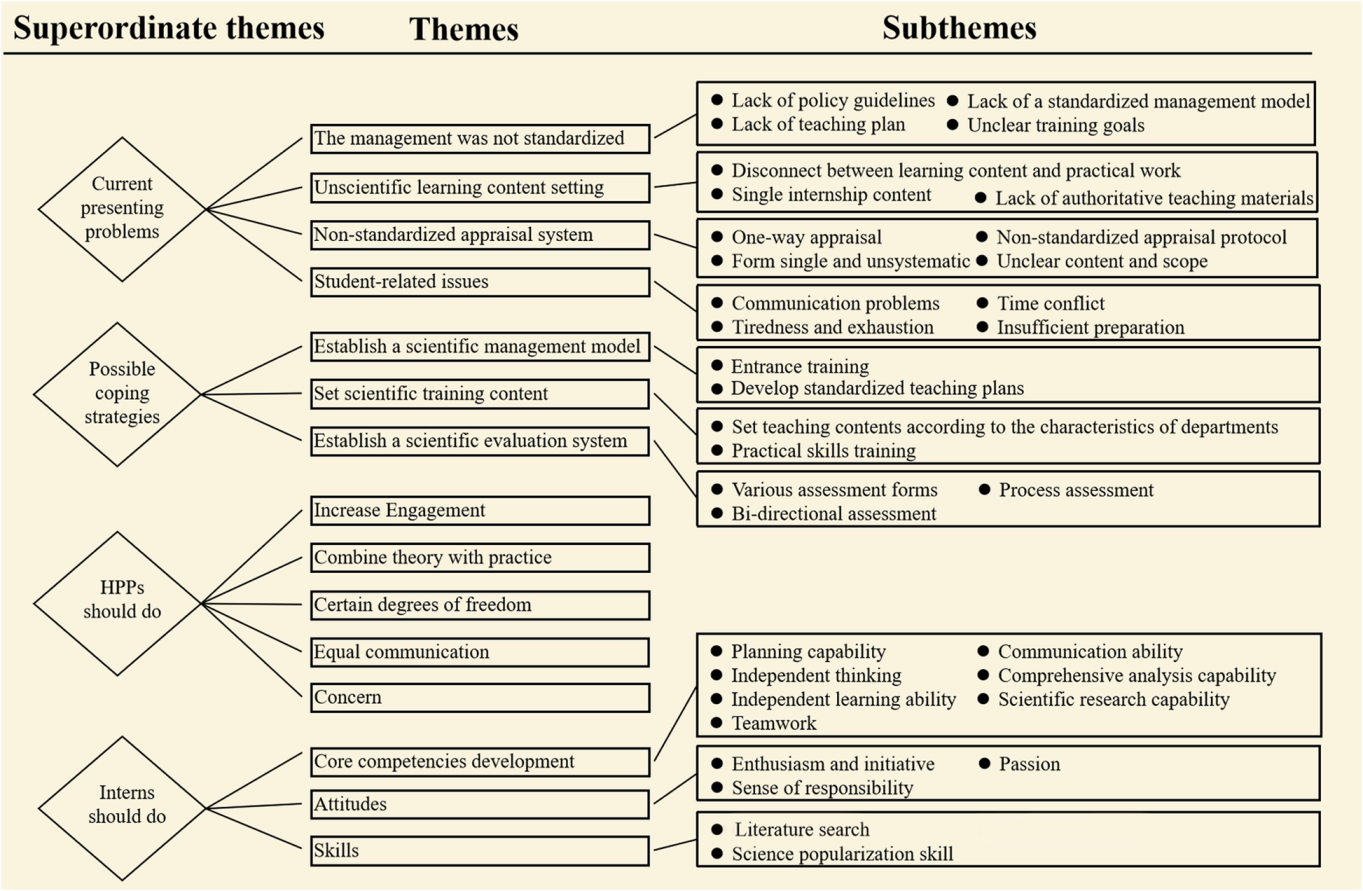
Thematic tree of HPPs’ experiences, challenges, and strategies for interns teaching

The analysis resulted in four superordinate themes, 15 themes, and 34 subthemes. The four themes form current presenting problems to possible coping strategies and something HPPs should do, as well as something interns should do. The framework of superordinate themes, themes, and sub-themes is shown in Fig. 2.

#### Current presenting problems

As stated by participants, there were four current presenting problems: 1) The management was not standardized; 2) Unscientific learning content setting; 3) Non-standardized appraisal system; 4) Student-related issues. A summary of the current problems regarding interns' education from the perspective of HPPs is presented in Table S2.

##### The management was not standardized

The most mentioned by HPPs is that the management was not standardized, mainly reflected in the lack of policy guidance and lack of a standardized management model. Apart from these two macro-aspects, from the micro side, HPP pointed out that there is a lack of teaching plans and unclear training goals.

##### Unscientific learning content setting

HPPs in our study said that the setting of learning content was not scientific, which is mainly reflected in the disconnection with the actual work, the single content, the lack of authoritative teaching materials, etc.

##### Non-standardized appraisal system

As an important part of the internship, the assessment system is not standardized in the view of the HPPs. For example, the current assessment is mostly a one-way evaluation by teachers to assess students, and a relatively single assessment mode based on closed-book written examination, and it is not clear about the content and scope that students should master.

##### Student-related issues

HPPs also mentioned that there are some problems with the students, such as some students not communicating with HPPs and not giving feedback on their teaching, which affects the quality of teaching; some students have time conflicts between their work and hospital internship, which affects the effectiveness of their internship; some students feel fatigued due to the repetition of a single placement content for a long time; some students lack planning for their internship and future work.

#### Possible coping strategies

Three possible strategies for coping with current presenting problems were captured from the interviews: establish a scientific management model, set scientific training content, and establish a scientific evaluation system (Table S3).

##### Establish a scientific management model

In addressing the issue of non-standardized management, HPPs suggested that before the formal internship, students are trained for admission. Admission training is to let students understand the purpose, specific content, and related requirements of an internship in the hospital, which can be summarized to form a standardized teaching program.

##### Set scientific training content

In addition, when setting the training content, HPPs pointed out that it is necessary to combine the characteristics of different departments to set the teaching content and pay attention to practical skills training.

##### Establish a scientific evaluation system

Assessment is an important part of the construction of the internship system. HPPs considered that it should take various forms, such as theoretical assessment, clinical practice assessment, medical ethics assessment, etc. At the same time, process assessment was considered by the HPPs to make a dynamic evaluation of the student's ability level and quality during the internship. Finally, the participants believe that it is necessary to change the past practice of only testing students' "learning" and ignoring teachers' "teaching", and to implement bi-directional assessment, to truly and objectively grasp the problems in teaching and learning, timely correct or adjust teaching plans, and promote the quality of teaching.

#### Something HPPs should do

In the process of pharmacy internship, HPPs are in the leading position. The quality of pharmacy internship teaching mainly depends on the level of HPPs teaching. The summary of “something HPPs should do” is presented in Table S4.

First of all, in the view of HPPs, based on preliminary familiarity with the work content and process of each department, interns can be guided and supervised to complete each task alone, to improve their participation. HPPs believe that it can promote the combination of theory and practice to a certain extent. Secondly, HPPs believed that interns should be allowed to explore freely, which is conducive to the cultivation and development of interns' self-learning and innovation abilities. Thirdly, some participants pointed out that the relationship between HPPs and interns is equal and democratic. To educate students well, the first task is to create a relaxed and harmonious teaching atmosphere. HPPs should put down their stance, communicate more with students, make friends with them, and care for them in life and study so that interns can lay a solid foundation for going into society in an environment of equal communication and care, and love.

#### Something interns should do

Pharmacy internship also has certain requirements for interns. Those skills that are important in the view of HPPs and need to be mastered by interns are summarized and shown in Table S5.

##### Core competencies

In our study, HPPs pointed out that interns should have some abilities, such as planning ability, communication ability, independent thinking ability, comprehensive analysis ability, self-directed learning ability, scientific research ability, and teamwork ability. These abilities are recognized as hospital pharmacists’ core competence in previous studies.

##### Attitudes

In addition to core competence, HPPs believed that the interns’ attitude during the internship is also very important as the HPPs expect them to be enthusiastic and initiative and have a passion for what they are learning, as well as a sense of responsibility.

##### Skills

The skills of literature search and science popularization are also mentioned by HPPs. The former is the basic means for interns to obtain knowledge and information, and the latter is a basic way for interns to practice "bring science in the field of pharmacy to general society".

## Discussion

To the best of our knowledge, the present study is one of the first qualitative studies in China to explore the hospital pharmacy preceptors’ perceptions of interns' teaching. The results of this study revealed the existing problems in interns' teaching, such as non-standardized management, unscientific learning content setting, non-standardized appraisal system, and student-related issues. Meanwhile, proposed possible coping strategies, and drew out what specifically the HPPs and students should do from HPPs’ personal experiences. The findings of the present study provide references for future hospital pharmacy education reform and policy development.

Pharmacy interns are regarded as potential pharmacists. A study conducted by Zhang et al. showed that in the Chinese context, the career intention of more than half of the undergraduate pharmacy students was to work in public medical institutions; that is, they aspired to become hospital pharmacists. Despite this, there is an insufficient workforce of pharmacists [23]. In addition, the majority of pharmacists in China play the traditional role of dispensing medications and providing basic medication counseling and are not capable of providing competent pharmaceutical care. Therefore, how to cultivate these pharmacy students into qualified pharmacists who meet the needs of our country is a question we need to think about. In addition to the study of theoretical courses, a hospital pharmacy internship is critical for the transition from "pharmacy students" to "professional pharmacists" [24].

This study identified four major problems and corresponding solutions from HPPs’ perspective. Firstly, for the non-standard management mode, we need to establish one, preferably at the national level. In 2018, the Ministry of Education of China released *national standards for the teaching quality of undergraduate majors at regular colleges and universities*, which is the first national standard for teaching quality in pharmacy and clinical pharmacy training [25]. However, this national standard is only a bottom-line requirement, with no detailed description or requirement of the hospital pharmacy internship, looking forward to the next step of modification and improvement.

Secondly, for an unscientific setting of learning content, it is necessary to propose one scientific system. However, a good content system cannot be constructed all at once, it requires the joint efforts of HPPs, training bases, and especially interns. For example, the feedback of interns was obtained at the end of each training year, and used to further improve the internship schedule and content in a hospital-based pharmacy internship program in Jordanian universities [26]. In our previous study, we also obtained very useful opinions and suggestions from interns for the construction of content systems, such as taking the core competency model of pharmacists as a reference to design internship content [8]. In this study, HPPs offered their recommendations for setting training content, including setting training content according to the characteristics of departments and emphasizing practical skill training. A well-structured learning content provides interns exposure to multiple departments of the pharmacy and gradually increase the complexity of tasks and responsibility according to the characteristics of departments. Direct patient and healthcare-provider interaction further strengthens practical skills, such as communication and listening [24].

Thirdly, aiming at the problems existing in the assessment of interns, HPPs also gave some suggestions, such as diversifying the forms of assessment, the use of process assessment and bi-directional assessment, etc. Previous assessments of interns have mostly focused on written exams. Considering the importance of pharmacy practice, the assessment of practice should also be considered and can be carried out using a combination of assessment methods, such as written exams, competency-based written assessments, objective structured clinical examinations (OSCE), process assessments, etc. [27]. Among these, the OSCE was introduced by Professor Harden in the UK and has been used since the 1970s as an important tool for the assessment and training of clinical medical students in the UK and the US by providing an objective and structured examination framework that simulates clinical scenarios to test students' theoretical knowledge, clinical skills, and work attitudes [28, 29]. The OSCE concept can also be applied to simulate real-life situations of pharmacists' work to assess and evaluate the overall quality of interns. In addition, HPPs also propose process assessment, it’s a process of dynamic evaluation of the student's ability level and quality during the internship so that interns can understand their mastery of knowledge through process assessment feedback, improve their learning methods, and enhance their independent learning ability in time. However, the assessment of interns should be moderate, because too much evaluation seems to make them focus each rotation on how to pass the exam, rather than what they learn during the rotation [30]. Finally, HPPs proposed bi-directional assessment, that is, when teachers assess students, students also evaluate teachers, which is a two-way feedback process, and teachers need to give constructive feedback after evaluating students. A survey of HPPs showed that preceptors ranked communication skills, effective feedback, and clinical knowledge as the most important elements of being an effective preceptor [31]; the comments given by students when assessing teachers also help teachers to improve their teaching methods.

Finally, the present study is a continuation of our previous study [8], which explored the experiences and expectations of pharmacist interns from the perspective of interns, while this study analyzed the pharmacy internship from the perspective of HPPs. After comparison, we found that most of the items on the list that something teachers should do has been mentioned in our previous research, such as combining theory with practice, equal communication, and taking care of students. Increased interns' engagement and certain degrees of freedom were new finding points. In the list of what interns should do, core competencies development was mentioned again in this study, which shows the importance of these skills. In addition to core competencies, good attitudes such as enthusiasm, initiative, passion, and responsibility were identified by the HPPs as necessary for the interns during their internship, as well as some skills such as literature searching and science popularization.

After comparing these two studies, we found an interesting phenomenon that most of the interns' interviews talked about their expectations and suggestions for teachers, while most of the interviews with the HPPs talked about their expectations of interns. The two sides have not sufficiently explored their problems and combining the two studies can more fully show the problems and strategies existing in pharmacy internships.

As of now, our two studies have delved into internship experiences and preceptorship experiences from the perspectives of interns and preceptors, respectively. We have identified key factors such as teacher-student interaction, a more scientific preceptorship model, standardized preceptorship plans, and bidirectional assessment based on interaction. Future research will focus on a more in-depth exploration of the multidimensional factors influencing internship experiences and their interactions, further exploration of scientific preceptorship models, and the development of more detailed elements for preceptorship plans. Currently, we are conducting a longitudinal design study using a mixed-methods approach, divided into three phases, with the first phase already completed. To incorporate these research findings into internship and curriculum plans, we plan to formulate practical guidelines, develop training courses, implement these methods and courses in our hospital, and establish an effective feedback mechanism to evaluate the implementation outcomes.

The strengths and limitations of the present study have to be mentioned. This study is one of the first qualitative studies in China to explore the HPPs’ perceptions of interns' teaching. To make the results more generalizable, it included HPPs from three large teaching hospitals in representative provinces of mainland China. Furthermore, our study is a continuation of our previous study, which explored the experiences and expectations of pharmacist interns from the perspective of interns, combining the two studies can more fully show the problems and strategies existing in pharmacy internships. However, the sample size, although typical for qualitative studies, is relatively small compared to quantitative studies, potentially affecting the representation of the entire population. Another disadvantage was gender imbalance, with more female participants. So, in the future, it would be advisable to include a more gender-balanced sample of HPPs to mitigate these limitations.

## Conclusion

In conclusion, this study revealed the existing problems in interns' teaching, such as non-standardized management, unscientific learning content setting, non-standardized appraisal system, and student-related issues. Meanwhile, proposed possible coping strategies, and drew out what specifically the HPPs and students should do from HPPs' personal experiences, which could provide references for future hospital pharmacy internships and policy development.

### Supplementary Information

**Additional file 1:** **Table S1.** Consolidated criteria for reporting qualitative studies (COREQ): 32-item checklist. **Table S2.** Theme 1 Current presenting problems. **Table S3.** Theme 2 Possible coping strategies. **Table S4.** Theme 3 Something HPPs should do. **Table S5.** Theme 4 Something interns should do.

## Data Availability

The datasets used during the current study are available from the corresponding author upon reasonable request.
