# Supplementary material for: Exploring the problems and coping strategies of pharmacy internship in large general hospitals in China: from the perspective of preceptors
Source: BMC Med Educ. 2024 Jan 17;24:69. doi: 10.1186/s12909-024-05032-x (PMC10795210; doi:10.1186/s12909-024-05032-x)
Supplement: Supplementary file 1 — Additional file 1: Table S1. Consolidated criteria for reporting qualitative studies (COREQ): 32-item checklist. Table S2. Theme 1 Current presenting problems. Table S3. Theme 2 Possible coping strategies. Table S4. Theme 3 Something HPPs should do. Table S5. Theme 4 Something interns should do. [file 12909_2024_5032_MOESM1_ESM.docx]

**Table S1 Consolidated criteria for reporting qualitative studies (COREQ): 32-item checklist**

| No Item | Guide questions/description | Answers |
| --- | --- | --- |
| **Domain 1: Research team and reflexivity** | | |
| Personal Characteristics | | |
| 1. Interviewer/facilitator | Which author/s conducted the interview or focus group? | Zhao Yin, Xiaoyue Bao, and Wan Zhang |
| 2. Credentials | What were the researcher’s credentials? E.g. PhD, MD | Zhao Yin, MS; Xiaoyue Bao, MS; Wan Zhang, BS. |
| 3. Occupation | What was their occupation at the time of the study? | Hospital pharmacists |
| 4. Gender | Was the researcher male or female? | Zhao Yin, Male; Xiaoyue Bao, Female; Wan Zhang, Female. |
| 5. Experience and training | What experience or training did the researcher have? | Theoretical training and experience of conducting several qualitative studies with other groups. |
| Relationship with participants | | |
| 6. Relationship established | Was a relationship established prior to study commencement? | Yes |
| 7. Participant knowledge of the interviewer | What did the participants know about the researcher? e.g. personal goals, reasons for doing the research | Reasons for doing the research |
| 8. Interviewer characteristics | What characteristics were reported about the interviewer/facilitator? e.g. Bias, assumptions, reasons and interests in the research topic | Reasons and interests in the research topic |
| **Domain 2: study design** | | |
| Theoretical framework | | |
| 9. Methodological orientation and Theory | What methodological orientation was stated to underpin the study? e.g. grounded theory, discourse analysis, ethnography, phenomenology, content analysis | Phenomenology. |
| Participant selection | | |
| 10. Sampling | How were participants selected? e.g. purposive, convenience, consecutive, snowball | Purposive |
| 11. Method of approach | How were participants approached? e.g. face-to-face, telephone, mail, email | face-to-face or video chat. |
| 12. Sample size | How many participants were in the study? | 14 |
| 13. Non-participation | How many people refused to participate or dropped out? Reasons? | No one refused and dropped out. |
| Setting | | |
| 14. Setting of data collection | Where was the data collected? e.g. home, clinic, workplace | face-to-face or video chat. |
| 15. Presence of non-participants | Was anyone else present besides the participants and researchers? | No |
| 16. Description of sample | What are the important characteristics of the sample? e.g. demographic data, date | Yes, demographic data. |
| Data collection | | |
| 17. Interview guide | Were questions, prompts, guides provided by the authors? Was it pilot tested? | Yes |
| 18. Repeat interviews | Were repeat interviews carried out? If yes, how many? | No |
| 19. Audio/visual | Did the research use audio or visual recording to collect the data? | Audio recording |
| 20. Field notes | Were field notes made during and/or after the interview or focus group? | Yes |
| 21. Duration | What was the duration of the interviews or focus group? | 25-50 min |
| 22. Data saturation | Was data saturation discussed? | Yes |
| 23. Transcripts returned | Were transcripts returned to participants for comment and/or correction? | Yes |
| **Domain 3: analysis and findingsz** | | |
| Data analysis | | |
| 24. Number of data coders | How many data coders coded the data? | Two |
| 25. Description of the coding tree | Did authors provide a description of the coding tree? | Yes |
| 26. Derivation of themes | Were themes identified in advance or derived from the data? | Derived from the data |
| 27. Software | What software, if applicable, was used to manage the data? | NVIVO 12 |
| 28. Participant checking | Did participants provide feedback on the findings? | Yes |
| Reporting | | |
| 29. Quotations presented | Were participant quotations presented to illustrate the themes / findings? Was each quotation identified? e.g. participant number | Yes |
| 30. Data and findings consistent | Was there consistency between the data presented and the findings? | Yes |
| 31. Clarity of major themes | Were major themes clearly presented in the findings? | Yes |
| 32. Clarity of minor themes | Is there a description of diverse cases or discussion of minor themes? | Yes |

**Table S2: Theme 1 Current presenting problems**

| **Theme1** | **Current presenting problems** |
| --- | --- |
| **1.1** | **The management was not standardized** |
| 1.1.1 | Lack of policy guidelines |
|  | *At present there is still an overall lack of some more standardised guidelines on internship education.(G1P1)*  *I feel that there is currently no good policy guidance for hospital pharmacy education from a national level. There is also no systematic training programme at the individual hospital level, and most teaching is based on personal experience.(G1P2)* |
| 1.1.2 | Lack of a standardized management model |
|  | *In my experience, there is an overall lack of a more standardized management model，different departments of the pharmacy arrange interns to work on a casual basis and there is no overall planning.(P2)*  *There is no set pattern and is not standardized, everything is a lesson plan for each department, which I think is something that needs to be improved in the future.(G1P2)* |
| 1.1.3 | Lack of teaching plan |
|  | *The length of student internships varies and has a large impact on the teaching plan. (P3)* |
| 1.1.4 | Unclear training goals |
|  | *No one tells me what kind of goal the intern is supposed to achieve, so I felt it was not so clear.(P8)* |
| **1.2** | **Unscientific learning content setting** |
| 1.2.1 | Disconnect between learning content and practical work |
|  | *The jobs they take after graduation may not be related to their practice in clinical pharmacy.(G1P2)*  *After graduation, most of the interns tend to choose the hospital pharmacy and social pharmacy, so their future work was somewhat disconnected from the practice of clinical pharmacy.(G1P3)* |
| 1.2.2 | Single internship content |
|  | *We may only teach them some simple operations, such as handling samples, and they may learn it in a day or two, but they will stay in our department for a month, and then he keeps repeating the process and will find it boring. (P3)* |
| 1.2.3 | Lack of authoritative teaching materials |
|  | *I have not seen books in this area, just read some published papers, most of which focus on the humanities, will do reference, but not authoritative.(P8)* |
| **1.3** | **Non-standardized appraisal system** |
| 1.3.1 | One-way appraisal |
|  | *I think we need to evaluate the teachers as well.(P7)* |
| 1.3.2 | Non-standardized appraisal protocol |
|  | In my opinion, the assessment of interns is not particularly systematic and regular, nor is it particularly perfect, and there is no standardized process.*(P5)* |
| 1.3.3 | Form single and unsystematic |
|  | *Our department has not formed a complete system for the evaluation of trainee teachers.(P6)*  *The possible problem is that there is no written examination, so it seems less systematic.(G2P1)*  *The overall assessment is just a formality.(P3)* |
| 1.3.4 | Unclear content and scope |
|  | *It needs to be clear what are the basic standards that students need to achieve, what things they should master, what things they need to accomplish, and what are the higher standards.(G2P2)* |
| **1.4** | **Student-related issues** |
| 1.4.1 | Communication problems |
|  | *The student doesn't understand what the teacher wants him to do, but he doesn't communicate either, and the result is not what the teacher wants, in fact，it delayed the time of both sides.(P3)*  *There is no feedback when communicating with the student, he listens to whatever you tell him, there is just no feedback and the interaction ability is very poor.(P8)* |
| 1.4.2 | Time conflict |
|  | *Based on the teaching mode of our department, sometimes students' interest points will conflict with teachers' teaching arrangements during this period of time.(P6)*  *Maybe the hospital internship time conflicts with the job internship they find, and students are not in the mood to continue to learn something related to clinical practice.* |
| 1.4.3 | Tiredness and exhaustion |
|  | *Students get tired of doing the same experiment or prescription review work for a long time.(P6)* |
| 1.4.4 | Insufficient preparation |
|  | *I found that many interns had a lack of career planning for their future.(P1)*  *Some students are not very clear about their future plans, in fact, we are sometimes the same as students.(G2P2)* |

**Table S3: Theme 2 Possible coping strategies**

| **Theme 2** | **Possible coping strategies** |
| --- | --- |
| **2.1** | **Establish a scientific management model** |
| 2.1.1 | Entrance training |
|  | *Communicate with interns at a deeper level to understand what they really want to learn, and then make internship plans for them according to our conditions. (P5)*  *We should emphasize these things with them at the time of admission training, so that they can better adapt to the internship process. (G2P3)*  *For each batch of interns, I will do the admission training for half an hour to an hour, which mainly involves the basic situation of our hospital and the basic situation of the Department of Pharmacy. (G2P1)*  *In the course of admission training, we should understand the interests of students, for example, which department they want to study, what they want to learn and so on. (P1)* |
| 2.1.2 | Develop standardized teaching plans |
|  | *Have a standard plan that goes beyond the surface. (P6)*  *Make a detailed internship plan for the interns. For example, make a list of the content to be involved to form our teaching plan. (P8)* |
| **2.2** | **Set scientific training content** |
| 2.2.1 | Set teaching contents according to the characteristics of departments |
|  | *Teachers in each department are required to do relevant theoretical learning plans, plus some common basic work, such as prescription review and prescription review. (G2P1)* |
| 2.2.2 | Practical skills training |
|  | *Internship teaching content, such as prescription review, medication account, these are the interns should focus on learning content. (P5)* |
| 2.2.3 | Ability training |
|  | *In terms of internship and teaching, we attach great importance to the cultivation of interns' ability, which is very crucial. (G1P1)* |
| **2.3** | **Establish a scientific evaluation system** |
| 2.3.1 | Various assessment forms |
|  | *I think there should be a variety of assessments. (P2)*  *For example, the assessment of drug education can be carried out during daily rounds, and it is not necessary to wait until the final unified assessment. (P6)*  *On the one hand, the assessment content should include the basic knowledge of clinical pharmacy that they master when entering the course, and the other is to lead the teacher to make some assessment content according to the work done by the students during the internship. (P6)* |
| 2.3.2 | Process assessment |
|  | *When interns give medication guidance to patients, I will observe them and conduct on-site assessment. (G1P3)*  *After our daily rounds, we have a pharmaceutical care discussion, and then we usually let the interns speak, and in the process, we evaluate one of their learning. (G1P2)* |
| 2.3.3 | Bi-directional assessment |
|  | *Establish a status of teacher-student evaluation, which may be more appropriate than the administration's evaluation of teachers. (P3)* |

**Table S4: Theme 3 Something HPPs should do**

| **Theme 3** | **Something HPPs should do** |
| --- | --- |
| **3.1** | **Increase interns' engagement** |
|  | *In the process of clinical ward rounds, medication rearrangement, doctor's order review and medication plan formulation, the teacher involved the interns to guide our working mode and improve their clinical practice skills. (G1P1)*  *If they can ensure that they communicate with 3 to 5 patients every day, their communication ability is absolutely fine. (G1P2)* |
| **3.2** | **Combine theory with practice** |
|  | *They can apply what they learn in the classroom to the clinic. (G1P2)*  *After familiar with the catalog of common drugs in this department, I think it is more meaningful to go into the clinic more and think about pharmacological effects and indications. (P4)* |
| **3.3** | **Give interns certain degrees of freedom** |
|  | *Freedom! Is freedom, for rounds, cases and drug education students have questions, to give them freedom, let them find their own answers, do not understand we discuss together. (P4)* |
| **3.4** | **Equal communication** |
|  | *I think when you talk to interns, you need to be clear, articulate, patient. Because you may be a little unclear, they understand that there is a deviation, which will cause a lot of misunderstandings. (P6)*  *Because I think students are a disadvantaged group, we hope to have an equal relationship with students. (P8)* |
| **3.5** | **Care for interns** |
|  | *They have just stepped out of school into the society, there will be a psychological gap, this time more need teachers to understand them, maintain them. (P6)*  *Whether we are teachers who take care of them or manage them, we should show some care in our life. (P6)* |

**Table S5: Theme 4 Something interns should do**

| **Theme 4** | **Something interns should do** |
| --- | --- |
| **4.1** | **Core competencies development** |
| 4.1.1 | Planning Capability |
|  | *Do some planning and learning on your own. (P5)*  *Have a work plan every day. What should I do today? Of course, there may be some temporary work, there are some unexpected situations, then at this time we must divide things into primary and secondary, know what to complete first, and then what to complete. (P6)* |
| 4.1.2 | Communication Ability |
|  | *Interns need good communication skills, you may know a lot, but if you can't express it, it won't accomplish your purpose. (P2)*  *Yes, you should communicate in a timely manner. The ability to communicate is really important. (P3)*  *It is more necessary to cultivate interns' ability of expression or communication. (G1P3) Communication skills for many different roles, such as patients, doctors, nurses, teachers, and people of different ages. (G2P1)* |
| 4.1.3 | Independent Thinking |
|  | *I think it's important to think independently. (P2)*  *The ability to think independently and solve problems independently. (P3)*  *In my opinion, it is to cultivate students' thinking ability, such as how to think about B or C when talking about A. (P6)* |
| 4.1.4 | Comprehensive analysis capability |
|  | *For example, adverse reaction reporting is not a simple report, do you have your own ideas about some requirements in the reporting process, to judge whether it is an adverse reaction, this is a comprehensive process. (P6)* |
| 4.1.5 | Self-directed learning ability |
|  | *I think the general point is that students should have their own ability to learn. Because a lot of research work is uncertain, you need to look up something, read some articles, and then get inspired from it, and then extrapolate back to your own topic. On the other hand, they need to take the initiative to learn by themselves. (G1P2)* |
| 4.1.6 | Scientific research capability |
|  | *I think it is more important for students to improve their scientific research ability. (P8)*  *In my opinion, one of the most important abilities is to consult the literature and extract the important content from the literature. (G1P2)*  *One of the skills that needs to be improved most is the ability to consult, organize and summarize literature. (G1P1)* |
| 4.1.7 | Teamwork |
|  | *Students who come together can study together and have group discussions. (G1P2)* |
| **4.2** | **Attitudes** |
| 4.2.1 | Enthusiasm and initiative |
|  | *If an intern does not have a proactive attitude, the harvest may be very limited for him. (P5) Self-discipline and initiative of interns are very important to them. (G1P2)*  *I will emphasize to them to improve their subjective initiative. (G2P3)*  *It feels like they have to be proactive and think for themselves. (P3)* |
| 4.2.2 | Passion |
|  | *Especially in doing scientific research, you have to be patient, because this thing is both boring and long-term, and you don't know when the results will come out, so you have to have patience, and the passion to devote yourself to it. (P2)* |
| 4.2.3 | Sense of responsibility |
|  | *For example, how can he complete his work without such a great sense of responsibility and awareness, it is difficult. (P3)* |
| **4.3** | **Skills** |
| 4.3.1 | Literature search |
|  | *In my opinion, one of the most important abilities is to consult the literature and extract the important content from the literature. (G1P2)* |
| 4.3.2 | Science popularization skill |
|  | *As the main disseminators of pharmacy science information in the future, the popularization ability of pharmaceutical students is very important.(G2P2)* |
